# Beneficial Effects of Gagam-Palmultang on Scopolamine-Induced Memory Deficits in Mice

**DOI:** 10.1155/2018/3479083

**Published:** 2018-02-18

**Authors:** Yu Ri Kim, Min Young Kwon, Malk Eun Pak, So Hyun Park, Jin Ung Baek, Byung Tae Choi

**Affiliations:** ^1^Korean Medical Science Research Center for Healthy-Aging, Pusan National University, Yangsan 50612, Republic of Korea; ^2^Department of Korean Medicine, School of Korean Medicine, Pusan National University, Yangsan 50612, Republic of Korea; ^3^Department of Korean Medical Science, School of Korean Medicine, Pusan National University, Yangsan 50612, Republic of Korea; ^4^Graduate Training Program of Korean Medicine for Healthy-Aging, School of Korean Medicine, Pusan National University, Yangsan 50612, Republic of Korea

## Abstract

From text mining of Dongeuibogam, the 7 herbs in Palmultang can be considered effective candidates for memory enhancement. We sought to determine whether Gagam-Palmultang, comprising these 7 herbs, ameliorates scopolamine-induced memory impairment in mice, by focusing on the central cholinergic system and memory-related signaling molecules. Behavioral tests were performed after inducing memory impairment by scopolamine administration. The cholinergic system activity and memory-related molecules were examined in the hippocampus by enzyme-linked immunosorbent, western blot, and immunofluorescence assays. Gagam-Palmultang ameliorated scopolamine-induced memory impairment in the Morris water maze test, producing a significant improvement in the mean time required to find the hidden platform. Treatment with Gagam-Palmultang reduced acetylcholinesterase activity and expression in the hippocampus induced by scopolamine. The diminished phosphorylated phosphatidylinositide 3-kinase (PI3K), extracellular signal-regulated kinase (ERK), cAMP response element-binding protein (CREB), and mature brain-derived neurotrophic factor (mBDNF) expressions caused by scopolamine administration were attenuated by treatment with Gagam-Palmultang. This treatment also promoted neuronal cell proliferation in the hippocampus. Gagam-Palmultang has beneficial effects against scopolamine-induced memory impairments, which are exerted via modulation of the cholinergic system as well as the PI3K and ERK/CREB/BDNF signaling pathway. Therefore, this multiherb formula may be a useful therapeutic agent for diseases associated with memory impairments.

## 1. Introduction

Aging-related cognitive- and memory-deficit conditions, such as Alzheimer's disease (AD), are becoming a public health problem as the lifespan of humans is increasing in the modern industrial society [1, 2]. The central cholinergic system is important in cognitive function and deficits in this system are linked to conditions such as aging-induced dementia, AD, and other neurodegenerative diseases [3–5]. The acetylcholine (ACh) that is required to control multiple cognitive processes is synthesized and hydrolyzed by choline acetyltransferase and acetylcholinesterase (AChE) in cholinergic neurons, respectively [6, 7]. Therefore, current therapeutic drugs for treatment of dementia and AD are based on the cholinergic system, such as cholinesterase inhibitors [8].

Brain-derived neurotrophic factor (BDNF) is strongly linked to mechanisms underlying consolidation of recognition memory, and modulation of this neurotrophic factor also affects cognitive function [9–11]. BDNF stimulates intracellular signaling cascades implicated in the molecular mechanisms of learning and memory enhancement [11]. Activation of downstream signaling pathways by BDNF, including the phosphatidylinositide-3-kinase (PI3K)/protein kinase B (Akt) and extracellular-signal-related kinase (ERK)/mitogen-activated protein kinase (MAPK) pathways that are known to activate transcriptional factor cAMP response element-binding protein (CREB), subsequently enhances cognitive function [12–14].

Among many approaches aimed at developing novel drugs for cognitive and memory impairment are certain herbs and multiherb formulae; some studies have focused on the use of these herbal drugs as a clinical therapy for such deficits [15, 16]. Palmultang is a traditional herbal medicine that contains 8 herbs; it has been known to supplement deficient qi and xue (blood) in the body, according to Oriental Medicine theory; thus this formula has been used to treat dizziness, dim eyesight, lassitude of limbs, and others symptoms associated with body weakness due to chronic illness [17]. A search of the Korean traditional medical literature Dongeuibogam for cognitive-enhancing medicinal herbs provided candidate therapeutic herbs than can be used to develop more effective therapeutic agents. Based on this text mining, Palmultang was found to contain 7 herbs considered effective for cognitive enhancement [18].

Palmultang shows significant inhibitory effects on the secretion of inflammatory factors in RAW 264.7 cells [19] and Palmul-Chongmyeong-tang consisting of 10 herbs has a protective effect against ischemia-induced neuronal and cognitive impairments in rats [20]. Gagambang (modified decoction) is a clinical practice that changes the composition or dosage of an existing prescription in order to enhance the therapeutic efficacy for certain symptoms and to minimize side effects in Korean medicine [21]. The blockade of muscarinic action by scopolamine, a nonselective a muscarinic cholinergic antagonist, is considered as a pharmacologic model for cognitive impairment [22].

Therefore, in the present study, we prepared Gagam-Palmultang, composed of 7 herbs, in order to enhance the therapeutic efficacy of the decoction for memory impairment. We evaluated the beneficial effects of Gagam-Palmultang on scopolamine-induced memory impairment in mice. Furthermore, we researched the beneficial effects of this multiherb formula on the central cholinergic system and memory-related signaling molecules, such as PI3K/Akt, ERK, CREB, and BDNF, in the hippocampus.

## 2. Materials and Methods

### 2.1. Chemicals and Antibodies

Scopolamine hydrobromide (P5295-25G) and piracetam (S1875-5G) were purchased from Sigma-Aldrich Corporation (St. Louis, MO, USA). The Amplex® Red Acetylcholine/Acetylcholinesterase Assay Kit (A12217) was purchased from Molecular Probes™ (Eugene, OR, USA). For western blot analysis, actin (A2066) was purchased from Sigma-Aldrich Corporation. ERK (sc-94) and Akt (sc-168) were supplied by Santa Cruz Biotechnology (Santa Cruz, CA, USA). Antibodies against phosphorylated ERK (pERK, #9101), PI3K (#4257), phosphorylated PI3K (pPI3K, #4228), CREB (#9197), phosphorylated CREB (pCREB, #9196), and phosphorylated Akt (pAkt, #4058) were supplied by Cell Signaling Technology (Danvers, MA, USA). For immunohistochemical analysis, antibodies against pPI3K (ab61801, Y607), mature BDNF (mBDNF, ab75040), and Ki67 (ab15580) were supplied by Abcam (Cambridge, UK). The antibody against neuronal nuclei (NeuN, MAB377) was supplied by Millipore Corporation (Billerica, MA, USA), and that against pCREB (sc-7978) was supplied by Santa Cruz Biotechnology. The antibody against AchE (PA5-21371) was supplied by Thermo Fisher Scientific (Waltham, MA, USA). Fluorescein anti-rabbit (FI-1000), fluorescein anti-sheep (FI-6000), and Texas Red anti-mouse (TI-2000) antibodies were purchased from Vector Laboratories Inc. (Burlingame, CA, USA).

### 2.2. Animals

**Figure 1:**
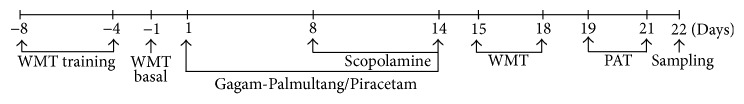
Schematic diagram of the overall experiment and behavioral testing. WMT, Morris water maze test; PAT, passive avoidance test.

C57BL/6 mice (aged 8 weeks, male, weight 25–30 g) were obtained from Dooyeol Biotech (Seoul, Korea). Mice were housed at 22°C under an alternating 12-h light/dark cycle; they were fed a commercial diet and allowed tap water ad libitum throughout the study. All experimental protocols were approved by the Pusan National University Animal Care and Use Committee in accordance with the National Institutes of Health Guidelines (approval number: PNU-2015-0774). A total of 43 mice were randomly divided into 6 groups as follows: control mice (*n* = 6), mice given a high dose of Gagam-Palmultang (PH, *n* = 7), mice administered scopolamine with vehicle (vehicle, *n* = 7), mice administered scopolamine with a low dose of Gagam-Palmultang (S + PL, *n* = 8), mice administered scopolamine with a high dose of Gagam-Palmultang (S + PH, *n* = 8), and mice administered scopolamine with piracetam as a positive control (S + Pira, *n* = 7). All experiments were performed as shown in the schematic diagram in Figure 1.

### 2.3. Preparation of Gagam-Palmultang

The composition of Gagam-Palmultang was based on the Dongeuibogam. Herbs were obtained from Pusan National University Korean Medicine Hospital (Yangsan, Korea) and were authenticated by Professor J.U. Baek, Department of Korean Medicine, School of Korean Medicine, Pusan National University. Gagam-Palmultang (*Panax ginseng* C. A. Meyer, 15.5 g,* Atractylodes macrocephala* Koidzumi 15.5 g,* Poria cocos* Wolf 15.5 g,* Glycyrrhiza uralensis* Fischer 15.5 g,* Angelica gigas* Nakai 15.5 g,* Rehmannia glutinosa* Liboschitz 15.5 g, and* Paeonia lactiflora* Pallas 15.5 g; total herbs 108.5 g) was immersed in 1,500 ml of water and extracted for 2 h 30 min by heating. The extract was subsequently filtered through Whatman No. 2 paper (Advantech, Milpitas, CA, USA), twice, and evaporated under reduced pressure using a vacuum evaporator (N-1000V-W, Eyela Co., Ltd., Tokyo, Japan). The final amount of extracted Gagam-Palmultang was about 26.6 g. The yield of Gagam-Palmultang was 24.5%.

### 2.4. Administration of Scopolamine, Piracetam, and Gagam-Palmultang

Scopolamine was dissolved in phosphate-buffered saline (PBS) and piracetam in 0.9% saline solution. Scopolamine was administered by intraperitoneal injection (1 mg/kg/day, 100 *μ*l/day) during days 8–14 of the experiment. Piracetam (200 mg/kg/day) and low dose (1 g/kg/day) and high dose (2 g/kg/day) Gagam-Palmultang were orally administered during days 1–14 of experiment. All the drugs were administered in a total volume of 100 *μ*l/day between 9 and 10 A.M. every day. Piracetam was used as positive control.

### 2.5. Morris Water Maze Test

The Morris water maze test was performed with slight modifications to evaluate spatial learning and memory [23, 24]. In the Morris water maze test, mice were trained to find the platform from 8 to 4 days before administering drugs. After about 3 days of relaxation, basal recording was performed at −1 days before administering drugs. After administering drugs on days 15–18 of the experiment, the final recordings were performed for another 4 days. The 5 trials were conducted per day, and the mice were allowed to rest for about 10 min between the trials. The tank (100-cm diameter and 50-cm height) was filled with water maintained at about 22 ± 3°C, and the water was dyed it white. A platform was placed halfway between the center and the edge of the tank. The platform was placed 0.5 cm beneath the surface of the water, and its position remained unchanged throughout this experiment. The test was continued until the mouse reached the platform or had lasted for 180 s. The results of this experiment were recorded and analyzed using SMART 2.5.18 software (Panlab S.L.U., Barcelona, Spain).

### 2.6. Passive Avoidance Test

A passive avoidance test was used with slight modifications to evaluate short-term memory using a manual avoidance chamber and based on the preference of mice for dark zone [25, 26]. The shuttle box in the chamber was divided into 2 zones, an illuminated zone and a dark zone, separated by a guillotine door. This test was conducted for a total of 3 days, and was conducted only once. Mice were trained for 2 days before the final recording. On day 1, we observed that, when a mouse was placed on the starting spot, where the light was always on, it moved to the opposite position, which was darker. On day 2, mice received an electric shock lasting 3 s (0.8 mA) when they arrived in the dark place and closed the door. On day 3, we recorded entry latency within 5 min to assess whether mice could recognize and remember the electric shock in the dark place. No electric shock was delivered during the final recording.

### 2.7. ACh and AChE Activity

Using the Amplex Red Acetylcholine/Acetylcholinesterase Assay Kit, we evaluated the activation of the neurotransmitter ACh and AchE activity in the brain, which are important for cognition and memory. In this assay, we first lysed the mouse hippocampi using RIPA buffer. The working solutions consisted of 400 *μ*M Amplex Red reagent containing 2 U/ml horseradish peroxidase (HRP), 0.2 U/ml choline oxidase, 1 U/ml acetylcholinesterase, or 100 *μ*M acetylcholine. The reaction began when 100 *μ*l working solution was placed in wells containing 100 *μ*l of prepared samples or a positive control. Samples were incubated for 1 h 30 min in the dark. The fluorescence was then measured using a SpectraMAX 190 spectrometer (Molecular Devices, Sunnyvale, CA, USA) (excitation wavelength: 571 nm; emission wavelength: 585 nm). The background fluorescence was corrected by subtracting the values derived from the no-acetylcholine and acetylcholinesterase control.

### 2.8. Western Blot Analysis

Hippocampus tissues were homogenized in lysis buffer containing 250 mM NaCl, 5 mM EDTA, 25 mM Tris-HCl (pH 8.0), 1% NP40, 1 mM PMSF, 10 mM NaF, 0.1 mM Na_3_VO_4_, and a protease inhibitor cocktail. Equal amounts of proteins were separated on 10–12% sodium dodecyl sulfate-polyacrylamide gels and then transferred onto nitrocellulose membranes (Whatman, Dassel, Germany) at 90 mA for 1 h. The membranes were incubated with primary antibodies overnight at 4°C. The membranes were subsequently incubated with horseradish peroxidase-conjugated secondary antibodies for 1 h. *β*-Actin was used as a loading control for all experiments. Specific bands were analyzed using an enhanced chemiluminescence system and ImageQuant LAS-4000 imaging system (Fujifilm Cor., Tokyo, Japan).

### 2.9. Immunohistochemistry

Frozen sections (25 *μ*m, coronal sections) were treated with 4% paraformaldehyde solution for 15 min. After washing with PBS, sections were incubated in a blocking buffer containing 0.3% Triton X-100, 5% normal serum, and 1x PBS for 1 h at room temperature. Sections were then incubated overnight at 4°C with primary antibodies. After washing with PBST, the sections were incubated with the fluorescent secondary antibody for 2 h at room temperature, in the dark, and washed 3 times with PBST. DAPI (Invitrogen, Eugene, OR, USA) staining was performed for 30 min. Slides were again washed with PBST and were then mounted with mounting medium (Dako North America Inc., Carpinteria, CA, USA) and images were captured using a fluorescence microscope (Carl Zeiss, Inc., Gottingen, Germany).

### 2.10. Statistical Analyses

All data were expressed as mean ± SEM and analyzed using the SigmaStat statistical program Version 11.2 (Systat Software, San Jose, CA, USA). The results were analyzed statistically using one-way ANOVA or repeated one-way ANOVA with Tukey's post hoc test for comparing more than 2 groups. A value of *P* < 0.05 was considered statistically significant.

## 3. Results

### 3.1. Effect of Gagam-Palmultang on Memory-Related Behaviors

**Figure 2:**
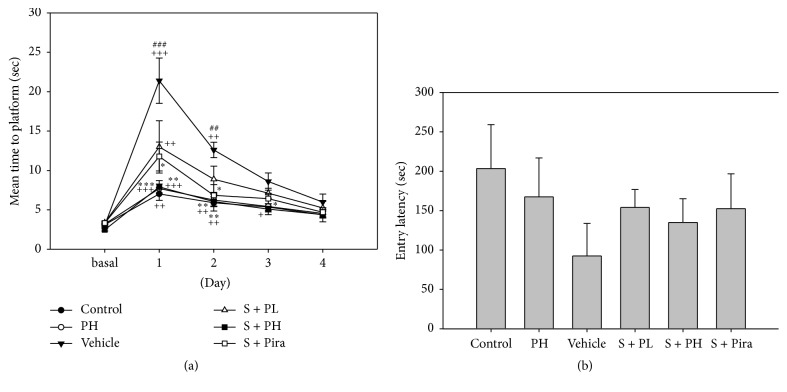
Effect of Gagam-Palmultang on memory-related behaviors. (a) Morris water maze test. (b) Passive avoidance test. Treatment with Gagam-Palmultang improves spatial learning and memory impairments caused by scopolamine. Data are presented as mean ± SEM. ^+^*P* < 0.05, ^++^*P* < 0.01, and ^+++^*P* < 0.001 versus basal; ^##^*P* < 0.01 and ^###^*P* < 0.001 versus control; ^*∗*^*P* < 0.05, ^*∗∗*^*P* < 0.01, and ^*∗∗∗*^*P* < 0.001 versus vehicle.

In the Morris water maze test for evaluating spatial memory, the vehicle group showed the highest mean time to platform value, as compared to baseline values (measured before treatment). The S + PL, S + PH, and S + Pira mice demonstrated a shorter latency to finding the platform than vehicle-treated mice. In particular, the S + PH and S + Pira mice showed a significantly shorter mean time for reaching the platform than did vehicle-treated mice [Figure 2(a)]. In the passive avoidance test for valuating short-term memory, vehicle mice exhibited a shorter entry latency than control mice; scopolamine-administered mice treated with Gagam-Palmultang, or with piracetam, exhibited a slightly longer latency, but there were no significant differences in the entry latency [Figure 2(b)]. These data suggested that treatment with Gagam-Palmultang had a beneficial effect on spatial memory impairment resulting from administration of scopolamine.

### 3.2. Effect of Gagam-Palmultang on the ACh and AChE Activity

**Figure 3:**
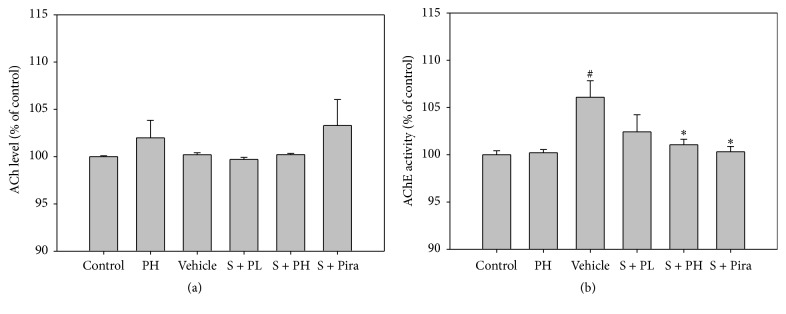
Effect of Gagam-Palmultang on ACh and AChE activity in the hippocampus. (a) ACh level. (b) AChE activity. The activity of AChE is significantly decreased by treatment with Gagam-Palmultang. Data are presented as mean ± SEM. ^#^*P* < 0.05 versus control; ^*∗*^*P* < 0.05 versus vehicle.

**Figure 4:**
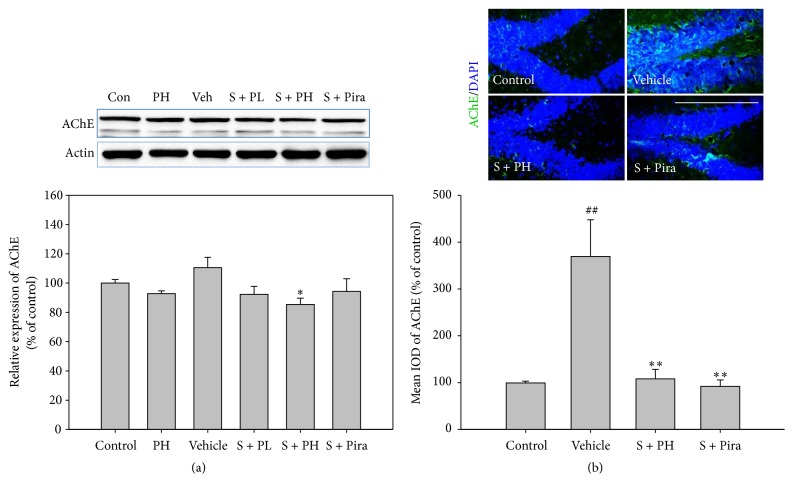
Effect of Gagam-Palmultang on AChE expression in the hippocampus. (a) Western blot and its histogram. (b) Photomicrograph for AchE/DAPI staining and its histogram. Compared to vehicle-treated mice, S + PH mice tend to show significantly decreased AChE expression. ^##^*P* < 0.01 versus control; ^*∗*^*P* < 0.05 and ^*∗∗*^*P* < 0.01 versus vehicle. Scale bar: 200 *μ*m.

We analyzed the ACh and AChE activity in the hippocampus. Although there were no significant differences among vehicle-treated and Gagam-Palmultang- and piracetam-treated mice in terms of ACh level, elevation of AChE activity by scopolamine was significantly reduced in the S + PH and S + Pira mice as compared to vehicle-treated mice (Figure 3). To confirm AChE activity in the hippocampus, we performed western blot and immunohistochemical analysis. The protein level of AChE generally tended to decrease in Gagam-Palmultang- and piracetam-treated mice as compared to vehicle-treated mice; in particular, there was a marked difference in the S + PH mice [Figure 4(a)]. The mean integrated optical density (IOD) of the AChE also showed a significantly lower value in S + PH and S + Pira mice than in vehicle-treated mice [Figure 4(b)]. These data suggested that treatment with Gagam-Palmultang produces significant attenuation of AChE activity in the hippocampus after scopolamine administration.

### 3.3. Effect of Gagam-Palmultang on Memory-Related Signaling Molecules

**Figure 5:**
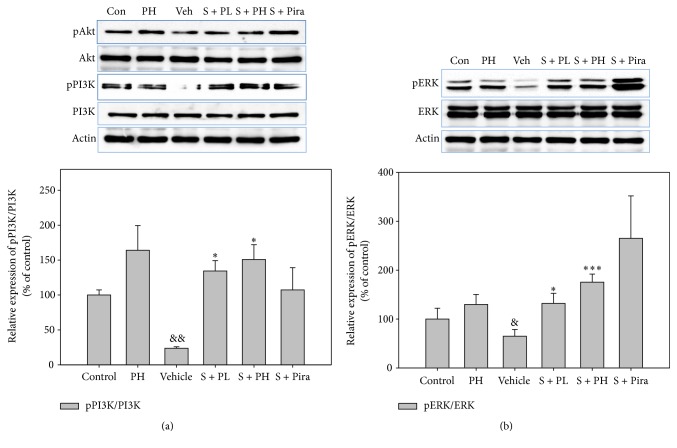
Effect of Gagam-Palmultang on the protein level of PI3K, Akt, and ERK in the hippocampus. (a) Western blot for PI3K and Akt and its histogram. (b) Western blot for ERK and its histogram. Protein levels of pPI3K and pERK are significantly increased by treatment with Gagam-Palmultang. Data are expressed as mean ± SEM. ^&^*P* < 0.05 and ^&&^*P* < 0.01 versus PH; ^*∗*^*P* < 0.05 and ^*∗∗∗*^*P* < 0.001 versus vehicle.

**Figure 6:**
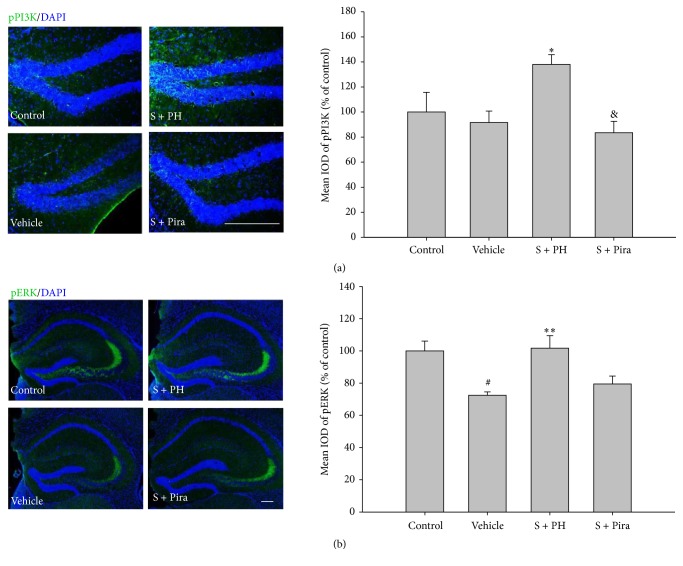
Effect of Gagam-Palmultang on the expression of pPI3K and ERK in the hippocampus. (a) Photomicrograph for pPI3K/DAPI staining and its histogram. (b) Photomicrograph for pERK/DAPI staining and its histogram. Expression of pPI3K and pERK is significantly increased by treatment with Gagam-Palmultang. Data are expressed as mean ± SEM. ^#^*P* < 0.05 versus control; ^*∗*^*P* < 0.05 and ^*∗∗*^*P* < 0.01 versus vehicle; ^&^*P* < 0.05 versus S + PH. Scale bar: 200 *μ*m.

Because PI3K/Akt and ERK are involved in multiple mechanisms related to memory, we investigated the activation of these proteins in the hippocampus. Protein levels of pPI3K, pAkt, and pERK were lower in vehicle-treated mice than in control mice, but recovered in scopolamine-administered mice treated with Gagam-Palmultang or piracetam. In particular, pPI3K and pERK levels in the S + PL and S + PH mice were markedly higher than those in vehicle-treated mice (Figure 5). The mean IODs of pPI3K and pERK in the hippocampus were also significantly higher in S + PH mice than those in the vehicle-treated mice (Figure 6).

**Figure 7:**
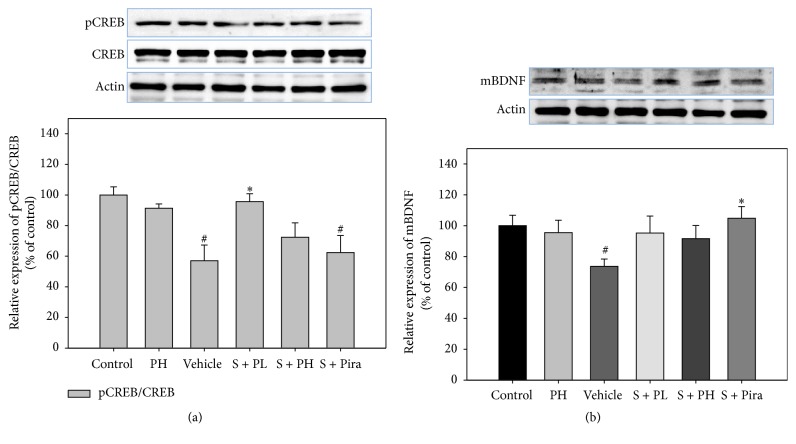
Effect of Gagam-Palmultang on the protein levels of CREB and mBDNF in the hippocampus. (a) Western blot for CREB and its histogram. (b) Western blot for mBDNF and its histogram. Protein levels of pCREB are significantly increased by treatment with Gagam-Palmultang. Data are expressed as mean ± SEM. ^#^*P* < 0.05 versus control; ^*∗*^*P* < 0.05 versus vehicle.

**Figure 8:**
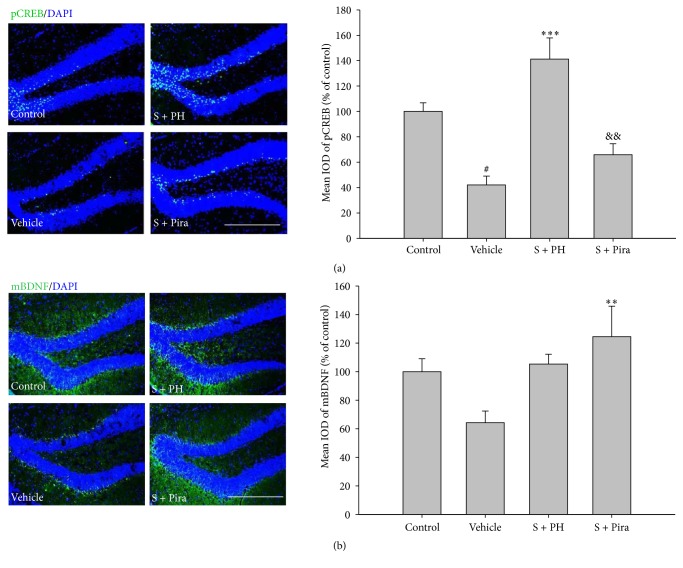
Effect of Gagam-Palmultang on the expression of pCREB and mBDNF in the hippocampus. (a) Photomicrograph for pCREB/DAPI staining and its histogram. (b) Photomicrograph for mBDNF/DAPI staining and its histogram. Expression of pCREB is significantly increased by treatment with Gagam-Palmultang. Data are expressed as mean ± SEM. ^#^*P* < 0.05 versus control; ^*∗∗*^*P* < 0.01 and ^*∗∗∗*^*P* < 0.001 versus vehicle; ^&&^*P* < 0.01 versus S + PH. Scale bar: 200 *μ*m.

To identify whether Gagam-Palmultang leads to activation of CREB and BDNF, we assessed the expression of the active forms of these proteins in the hippocampus. Vehicle-treated mice appeared to have markedly lower levels of pCREB and mBDNF than control mice, but pCREB and mBDNF levels were significantly increased in the S + PL and S + Pira mice, respectively (Figure 7). The mean IODs of pCREB and mBDNF were also significantly higher in S + PH and S + Pira mice than in vehicle-treated mice, respectively (Figure 8). These data suggested that Gagam-Palmultang may enhance activation of CREB through PI3K and ERK signaling molecules in the hippocampus after scopolamine administration, consequently leading to BDNF expression.

### 3.4. Effect of Gagam-Palmultang on Cell Proliferation

**Figure 9:**
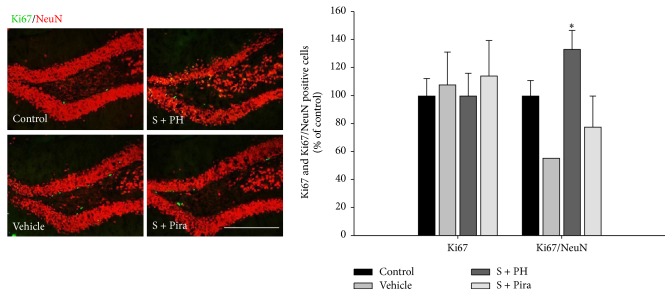
Effect of Gagam-Palmultang on the cell proliferation in the hippocampus. Photomicrograph for Ki67 or Ki67/NeuN staining and its histogram. The numbers of Ki67^+^/NeuN^+^ cells in the hippocampus tend to increase after treatment with Gagam-Palmultang. ^*∗*^*P* < 0.05 versus vehicle. Scale bar: 200 *μ*m.

Finally, we examined whether Gagam-Palmultang enhances cell proliferation in the hippocampus. The levels of the cell proliferation marker Ki67 were not significant in scopolamine-administered mice as compared to control mice. However, a significant increase in Ki67^+^/NeuN^+^ cells was detected in S + PH mice as compared to vehicle-treated mice (Figure 9). These data suggested that Gagam-Palmultang may enhance neuronal cell proliferation in the hippocampus after scopolamine administration.

## 4. Discussion

Defects in the central cholinergic system are involved in human memory disorders. ACh, a key component of the cholinergic system, is synthesized from acetyl-CoA and choline by choline acetyltransferase at cholinergic synapses. The duration of ACh signaling, which is engaged in learning and memory, is influenced by the activity of AChE, which hydrolyzes ACh to acetate and choline [3, 27]. Thus, injuries of these neurons are related to reductions in ACh levels and excessively increased AChE activity in neurodegenerative diseases, such as AD, and contribute to learning and memory dysfunction [3, 4]. Therefore, AChE inhibitors are potentially useful agents in the treatment of learning and memory impairments [28].

Administration of scopolamine blocks ACh receptors and is strongly associated with cognitive deficits in healthy humans and rodents [21]. In particular, scopolamine impairs spatial learning and memory in Morris water maze performance and is related to reductions in ACh and increased AChE activity in the hippocampus [29, 30]. As shown in previous studies, administration of scopolamine results in impaired learning and memory of the tasks involved in the Morris water maze test and in increased AChE activity in the hippocampus. However, treatment with Gagam-Palmultang ameliorated scopolamine-induced spatial learning and memory impairments, with restoration of the cholinergic system balance in the hippocampus, in this study. This result suggested that this formula may have therapeutic effects on cognitive impairments via restoration of the cholinergic system balance.

BDNF plays an important role in learning and memory, and activation of tyrosine receptor kinase B (TrkB) by this neurotrophin stimulates intracellular signaling cascades that are involved in mechanisms of cognitive enhancement [9, 11]. Binding of BDNF to TrkB subsequently triggers activation of 2 major signaling pathways, involving ERK/MAPK and PI3K/Akt [12–14, 31]. Activation of the ERK and PI3K/Akt pathways leads to expression of learning-related proteins, such as those involved in long-term potentiation [32–34]. Consequently, activation of signaling downstream of BDNF/TrkB further activates the transcription factor CREB [14]. Impairment of CREB phosphorylation is involved in a pathological component in conditions associated with memory impairment, such as AD, while pharmacologically induced CREB phosphorylation in the brain is associated with cognition [35].

We thus determined whether Gagam-Palmultang treatment affected memory-related signaling molecules in the scopolamine-induced model. Expression of hippocampal PI3K, ERK, CREB, and mBDNF decreased after scopolamine treatment. When we investigated the roles of Gagam-Palmultang in the activation of these molecules, we found that treatment with Gagam-Palmultang increased mBDNF levels in the hippocampus, according to western blot and immunofluorescence assays, accompanied by significant upregulation of pERK, pPI3K, and pCREB. This suggested that Gagam-Palmultang may improve the cognitive deficits in scopolamine-treated mice, and that these actions might be mediated by regulation of mBDNF levels in hippocampus via upregulation of PI3K/ERK/CREB.

The cholinergic system also affects neurogenesis in the hippocampus and plays an important role in learning and memory [36, 37]. An enhanced cholinergic system or AChE inhibition upregulates adult hippocampal neurogenesis through activation of neurogenic mechanisms, such as those involving BDNF and CREB [10, 36, 38]. Therefore, imbalance in the cholinergic system, that is, reduced Ach levels or increased AChE activity, induced by scopolamine could suppress cell proliferation in the hippocampus. We performed Ki67 immunohistochemistry to detect proliferative cells in the hippocampus. The number of Ki67-positive cells in the Gagam-Palmultang-treated mice was not significant as compared to scopolamine-treated mice. However, the number of Ki67/NeuN double-positive cells was higher in the Gagam-Palmultang-treated mice than in the scopolamine-treated mice, indicating that the scopolamine-induced loss of neuronal proliferation and differentiation was partially restored with Gagam-Palmultang treatment.

The question then arises as to whether any of the components of the Gagam-Palmultang could improve cognitive function. Previous studies have reported the simultaneous determination of 7 major components or 11 standard components in Palmultang [39, 40]. Several components, including hydroxymethylfurfural, liquiritin, albiflorin, paeoniflorin, ferulic acid, nodakenin, ginsenoside Rg1, decursinol, glycyrrhizin, 6-gingerol, ginsenoside Rg3, and decursin, have been separated and identified in this multiherb formula. Many of these components are associated with cognition. Hydroxymethylfurfural is a potential therapeutic agent for AD [41]; liquiritin and ferulic acid are useful for amelioration of amyloid *β* peptide-induced cognitive impairment [42, 43]; paeoniflorin reverses the muscarinic receptor-mediated inhibition of long-term potentiation [44]; nodakenin enhances adult hippocampal neurogenesis [45], ginsenoside Rg1 delays cognitive decline and promotes neurogenesis [46, 47], and glycyrrhizin ameliorates cognitive decline via anti-inflammatory effects [48]. Further studies of the efficacy and underlying mechanism of these Palmultang constituents should continue to elucidate their effects on cognitive dysfunction comprehensively.

## 5. Conclusion

In summary, scopolamine produced severe deficits in the performance of mice in the Morris water maze test, along with signs of memory impairments, including changed AChE activity and altered PI3K and ERK/CREB/BDNF pathways in the hippocampus. However, treatment with Gagam-Palmultang attenuated scopolamine-induced learning and memory deficits and restored the hippocampal cholinergic system balance. This treatment recovered the memory-related signaling involving PI3K and ERK/CREB/BDNF molecules. Therefore, our results suggest that Gagam-Palmultang ameliorates scopolamine-induced memory deficits via activation of PI3K and/or ERK/CREB/BDNF signaling. This multiherb formula may be a therapeutic agent for treatment of neurological disorders associated with cognitive impairments.
